# Cholesterol effects on the tumor immune microenvironment: from fundamental concepts to mechanisms and implications

**DOI:** 10.3389/fonc.2025.1579054

**Published:** 2025-04-09

**Authors:** Francisco Alejandro Lagunas-Rangel

**Affiliations:** ^1^ Department of Surgical Sciences, Uppsala University, Uppsala, Sweden; ^2^ Laboratory of Pharmaceutical Pharmacology, Latvian Institute of Organic Synthesis, Riga, Latvia

**Keywords:** SREBP2, LXR, LDLR, myeloid-derived suppressor cells, tumor-associated macrophages, statins

## Abstract

In many cancers, the tumor microenvironment is enriched with cholesterol due to increased biosynthesis and uptake by cancer cells, resulting in the accumulation of cholesterol, cholesterol esters, oxysterols and other metabolites with various functions. These molecules serve as structural components, energy sources and intracellular signaling mediators, while their toxic by-products are secreted to suppress anti-tumor immune activity and prevent lipid peroxidation that could induce cancer cell apoptosis. Immune cells in the tumor microenvironment also contribute to cholesterol dynamics. Tumor-associated macrophages (TAMs) release cholesterol to support tumor cell metabolism, while myeloid-derived suppressor cells (MDSCs) also release cholesterol and consume essential metabolites such as L-arginine, which impairs T-cell proliferation and activation. Elevated cholesterol in dendritic cells impairs migration and tumor antigen presentation and, in lymphocytes, favors the development of a regulatory T cells (Treg) phenotype and inhibits the release of antitumor cytokines, further weakening the immune response. These findings suggest that targeting cholesterol metabolism is a promising strategy for cancer treatment, improving the efficacy of immune checkpoint blockade (ICB) therapies. In this manuscript, the molecular mechanisms underlying the effects of cholesterol on the tumor immune landscape are reviewed and the potential of cholesterol-lowering drugs to enhance antitumor immune responses is explored.

## 1 Introduction

**Figure 1:**
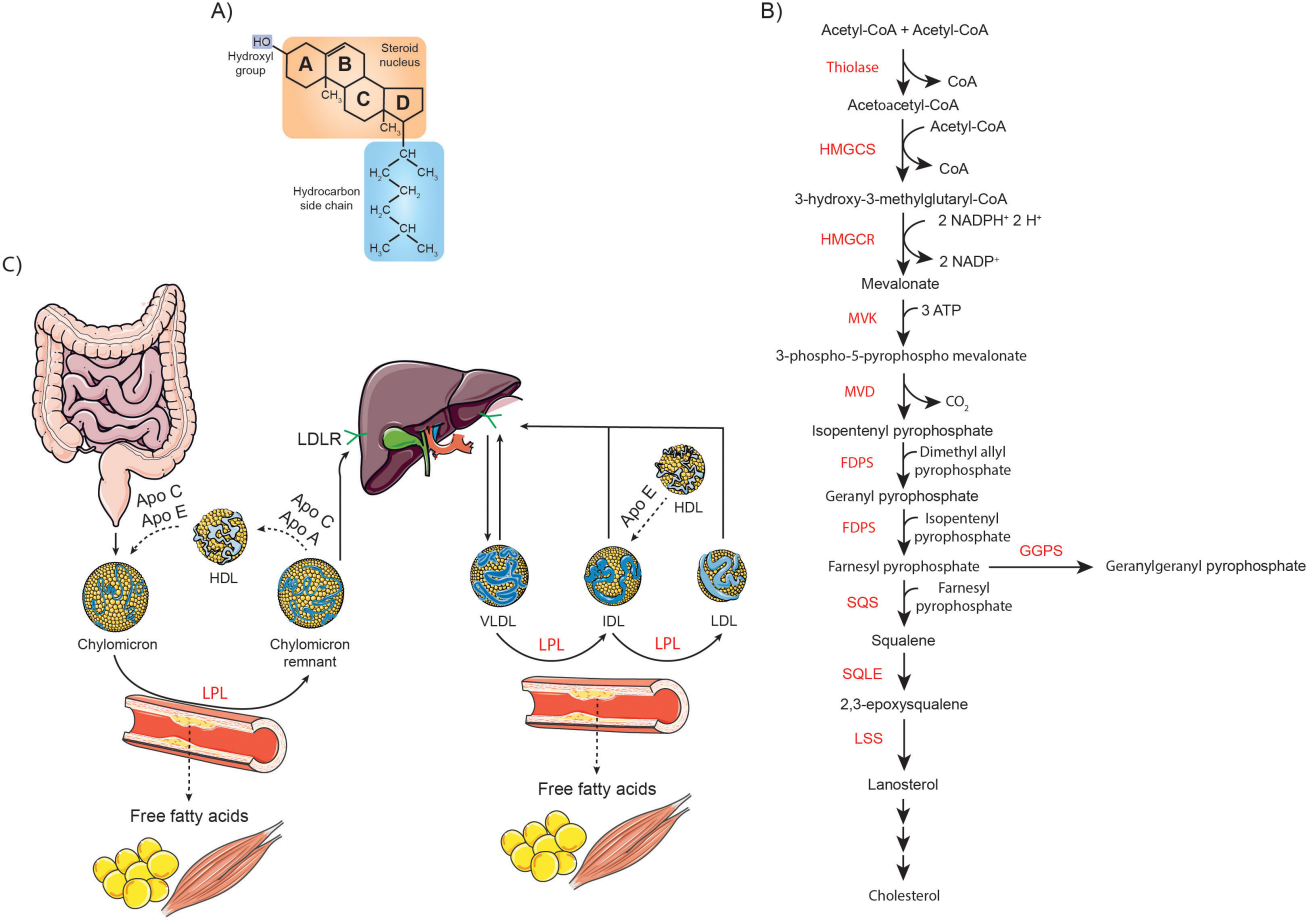
Overview of cholesterol structure, synthesis, and metabolism. **(A)** Schematic representation of the chemical structure of cholesterol, highlighting its characteristic sterol backbone and functional groups. **(B)** Diagram of the cholesterol biosynthetic pathway, detailing key enzymatic steps and intermediates involved in its synthesis. **(C)** Illustration of the absorption and systemic transport of dietary cholesterol, emphasizing the roles of intestinal uptake, incorporation into lipoproteins, and distribution throughout the body.

Cholesterol is an organic compound classified within the steroid family, characterized by its distinctive chemical structure: four fused hydrocarbon rings, which provide the molecule with extreme rigidity, a proximal hydrophilic hydroxyl (-OH) group at the 3-position, and a distal hydrophobic hydrocarbon tail extending from the 17-position (**Figure 1A**) (1). This unique architecture allows cholesterol to perform a wide range of cellular functions, such as regulating membrane fluidity and permeability, being a precursor of steroid hormones, corticosteroids, bile acids, and vitamins, as well as influencing gene transcription (2).

Cholesterol and its derivatives, including cholesterol esters and oxysterols, play several critical roles in cancer progression (3). Indeed, increased cholesterol biosynthesis is a hallmark of many types of cancer. Overall, both cancer cell intrinsic mechanisms and extrinsic signals reprogram cholesterol metabolism to support the structural and energetic demands of tumor progression and invasion. Cancer cells have an increased demand for cholesterol to maintain membrane biogenesis and other vital cellular processes (4). Notably, cholesterol is the main lipid component of lipid rafts, specialized microdomains of the plasma membrane that act as dynamic platforms for organizing signaling molecules. In cancer, lipid rafts regulate key events such as tumor cell growth, adhesion, migration, invasion and apoptosis, which ultimately determine cellular responses to external stimuli (5). Cholesterol precursors, farnesyl pyrophosphate (FPP) and its derivative geranylgeranyl pyrophosphate (GGPP), are key intermediates involved in the prenylation of oncogenic proteins such as RAS, which promote cancer cell survival and proliferation (6). Among cholesterol-derived metabolites, the oncometabolite 6-oxo-cholestan-3β,5α-diol binds to glucocorticoid receptors, promoting tumor growth (7). On the other hand, and the focus of this manuscript, metabolic reprogramming of cholesterol also dampens immune responses, creating a microenvironment favorable to tumor progression (8). Preclinical and clinical studies have shown that acting on cholesterol metabolism can effectively inhibit tumor growth, remodel the immune microenvironment, and reinvigorate antitumor immune responses (9). The aim of this work is to provide an overview of the impact of cholesterol on the tumor immune microenvironment, with particular attention to the underlying molecular mechanisms. Furthermore, the benefits of cholesterol-lowering drugs in enhancing antitumor immune responses and their potential to improve the efficacy of immune checkpoint blockade (ICB) therapies are examined. The novelty of this work lies in its comprehensive analysis of how cancer cells manipulate both their own cholesterol metabolism and that of surrounding cells to promote tumor growth. In turn, it examines how these alterations lead to elevated cholesterol levels in the tumor microenvironment, which suppress the antitumor activity of immune cells. Furthermore, unlike other similar studies, this work critically evaluates the concentrations of cholesterol and cholesterol-targeted drugs used in *in vitro* assays, comparing them to clinically relevant therapeutic doses to provide a more accurate perspective of their potential impact on cancer treatment.

## 2 Cholesterol sources

Cholesterol in the body comes from two main sources: it is either synthesized *de novo* by the cells or obtained from dietary intake, especially from foods of animal origin. Overall, cholesterol biosynthesis is a tightly regulated multistage process that takes place mainly in the liver, starting from acetyl-CoA as the precursor molecule. In the first stage, 3-hydroxy-3-methylglutaryl-CoA (HMG-CoA) synthase (HMGCS1 in the cytoplasm and HMGCS2 in the mitochondria) condenses two acetyl-CoA molecules to form acetoacetyl-CoA, which then reacts with a third acetyl-CoA to produce HMG-CoA. This intermediate is subsequently reduced to mevalonate by HMG-CoA reductase (HMGCR), the key rate-limiting enzyme in cholesterol synthesis. Mevalonate then undergoes a series of phosphorylation and decarboxylation reactions to produce isopentenyl pyrophosphate (IPP), an essential isoprene unit. Farnesyl pyrophosphate synthase (FDPS) catalyzes the sequential condensation of IPP with dimethylallyl pyrophosphate, forming geranyl pyrophosphate, and subsequently with another IPP molecule to produce FPP. Squalene synthase (SQS) then condenses two FPP molecules to form squalene. Squalene is oxidized by squalene monooxygenase (SQLE) to 2,3-epoxysqualene, which is cyclized to lanosterol by lanosterol synthase (LSS) (**Figure 1B**). Lanosterol then undergoes further enzymatic modifications via the Bloch pathway, the Kandutsch-Russell pathway or a hybrid of both, ultimately resulting in cholesterol (3, 10).

On the other hand, enterocytes absorb dietary cholesterol from the intestinal lumen through a process involving the cholesterol transporter Niemann-Pick C1-like protein 1 (NPC1L1). This process is facilitated by the clathrin adaptor protein NUMB and the adaptor protein LIM domain and actin-binding protein 1 (LIMA1), which help coordinate the internalization and transport of cholesterol into the enterocyte. Cholesterol is then actively transported to the liver in chylomicrons or in its free form, where it is metabolized. Once in the liver, oxidation occurs through enzymatic reactions, catalyzed predominantly by enzymes of the cytochrome P450 (CYP) family, as well as non-enzymatic reactions driven by reactive oxygen species (ROS), which result in the formation of oxysterols. Cholesterol is also converted to less harmful cholesteryl esters (CE) through the action of sterol O-acyltransferases (SOATs). Cholesterol and its derivatives are incorporated into very low-density lipoproteins (VLDLs), which are released into the bloodstream to facilitate their transport throughout the body. In peripheral tissues, VLDLs are metabolized by lipoprotein lipase (LPL), leading to the formation of intermediate density lipoproteins (IDLs). These IDL particles are relatively enriched in cholesterol esters. Through the subsequent action of LPL, IDL continue to release free fatty acids (FFA), eventually becoming low-density lipoproteins (LDL). Approximately 75% of circulating cholesterol is transported by LDL, while the remaining 25% is associated with high-density lipoproteins (HDL). Approximately 70% of circulating LDL is removed from the bloodstream through LDL receptor (LDLR)-mediated endocytosis in the liver, while the remaining LDL is taken up by extrahepatic tissues (**Figure 1C**) (11).

## 3 Cholesterol homeostasis

Cholesterol levels, both at the cellular and systemic levels, are tightly regulated by intricate mechanisms. The main transcriptional regulators responsible for the maintenance of cholesterol homeostasis are sterol regulatory element-binding protein 2 (SREBP2), liver X receptors (LXRs), and nuclear factor erythroid 2-related factor 1 (NRF1). When cholesterol or cholesterol-derived oxysterols accumulate, they deactivate the SREBP2 pathway by facilitating anchoring of the SREBP2-sterol regulatory element-binding protein cleavage-activating protein (SCAP) complex to the endoplasmic reticulum (ER) membrane. This process occurs through a sterol-dependent interaction between SCAP and insulin-induced gene protein (INSIG). This deactivation reduces cholesterol biosynthesis and uptake. At the same time, desmosterol, the immediate precursor of cholesterol in the Bloch biosynthetic pathway, together with oxysterols, binds to and activates LXRs, leading to up-regulation of genes involved in cholesterol efflux, such as ATP-binding cassette sub-family A member 1 (ABCA1), ATP-binding cassette sub-family G member 1 (ABCG1), ATP-binding cassette sub-family G member 5 (ABCG5) and ATP-binding cassette sub-family G member 8 (ABCG8), and others such as myosin regulatory light chain interacting protein (MYLIP). Elevated cholesterol levels also inhibit the nuclear translocation of NRF1, thus preventing its suppression of the LXR pathway. In contrast, under conditions of cholesterol deficiency, these regulatory systems act in a coordinated but opposing manner to increase cholesterol biosynthesis and uptake, while reducing cholesterol efflux and esterification, ensuring the restoration of cholesterol homeostasis.

## 4 Cholesterol effects on the tumor immune microenvironment

The tumor microenvironment consists of the non-cancerous host cells surrounding the tumor, such as fibroblasts, endothelial cells, neurons, adipocytes, and both adaptive and innate immune cells. In addition to these cellular components, the tumor microenvironment also includes non-cellular elements such as the extracellular matrix (ECM) and soluble factors such as chemokines, cytokines, growth factors and extracellular vesicles, all of which play a key role in tumor development and progression (12).

Fibroblasts contribute to tumor progression by secreting extracellular matrix components, growth factors and cytokines that promote tumor cell invasion, angiogenesis and immune modulation (13). Meanwhile, endothelial cells form the tumor vasculature, regulating blood flow, nutrient supply and facilitating metastasis by allowing the exchange of molecules between blood and tumor cells (14). Neurons, especially in tumors affecting the nervous system, release neurotransmitters and signaling molecules that can influence tumor cell behavior, including proliferation and survival (15). Adipocytes, as a key component of the tumor stroma, release lipids, cytokines and growth factors that contribute to inflammation, immune suppression and metabolic reprogramming of tumor cells (16).

Tumor-associated immune cells can generally be classified into two categories: tumor-antagonistic immune cells and tumor-promoting immune cells. Tumor antagonist immune cells include effector T cells (such as cluster of differentiation 8 [CD8^+^] cytotoxic T cells and CD4^+^ effector T cells), natural killer (NK) cells, dendritic cells, M1-type macrophages and N1-type neutrophils, which contribute to suppress tumor growth. In contrast, tumor-promoting immune cells consist mainly of Tregs, myeloid-derived suppressor cells (MDSCs), M2-type macrophages and N2-type neutrophils, which facilitate tumor progression by suppressing antitumor immune responses (17, 18).

One study reported that tumors with elevated cholesterol metabolism are associated with an immunosuppressive microenvironment, characterized by an increased presence of Tregs and M2-type macrophages. In contrast, tumors with low cholesterol metabolism show a higher abundance of CD8^+^ cytotoxic T cells and proinflammatory M1-type macrophages (19). Another cell type to be mentioned are innate lymphoid cells (ILC), ILC1, ILC2 and ILC3. These cells infiltrate solid tumors early in the immune response and can exert either antitumor or protumor effects. This dichotomy is due to the significant heterogeneity and plasticity observed among the different ILC subsets (20).

**Figure 2:**
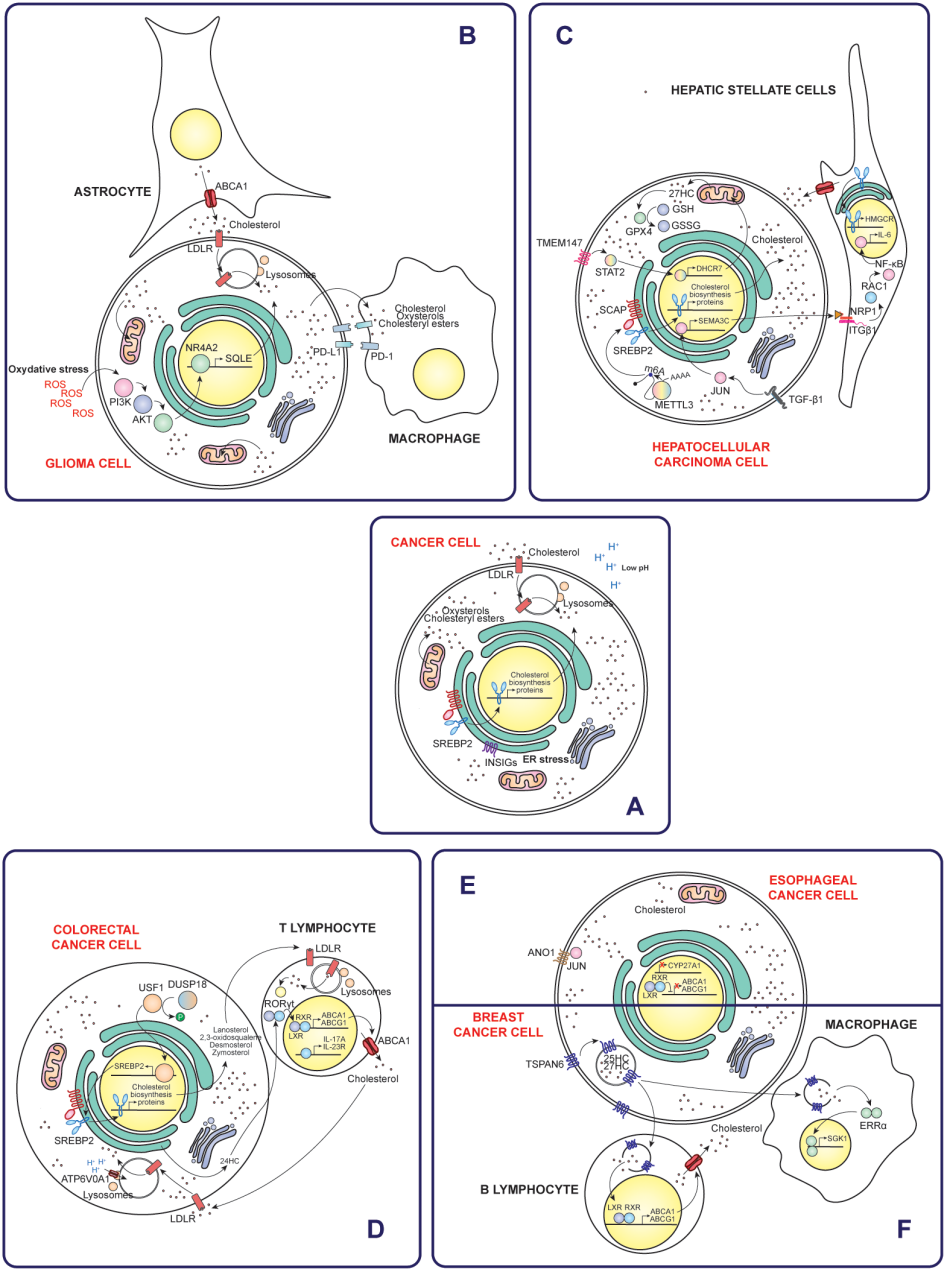
Mechanisms of cholesterol accumulation in cancer cells and their interactions with the immune tumor microenvironment. **(A)** General mechanisms by which cancer cells increase intracellular cholesterol levels, such as increased uptake, synthesis and reduced efflux. Specific examples of different types of cancer include gliomas **(B)**, hepatocellular carcinoma **(C)**, colorectal cancer **(D)**, esophageal cancer **(E)** and breast cancer **(F)**. Taken together, these mechanisms highlight the diverse strategies of cholesterol modulation in different types of cancer and their implications for the immune tumoral microenvironment.

**Figure 3:**
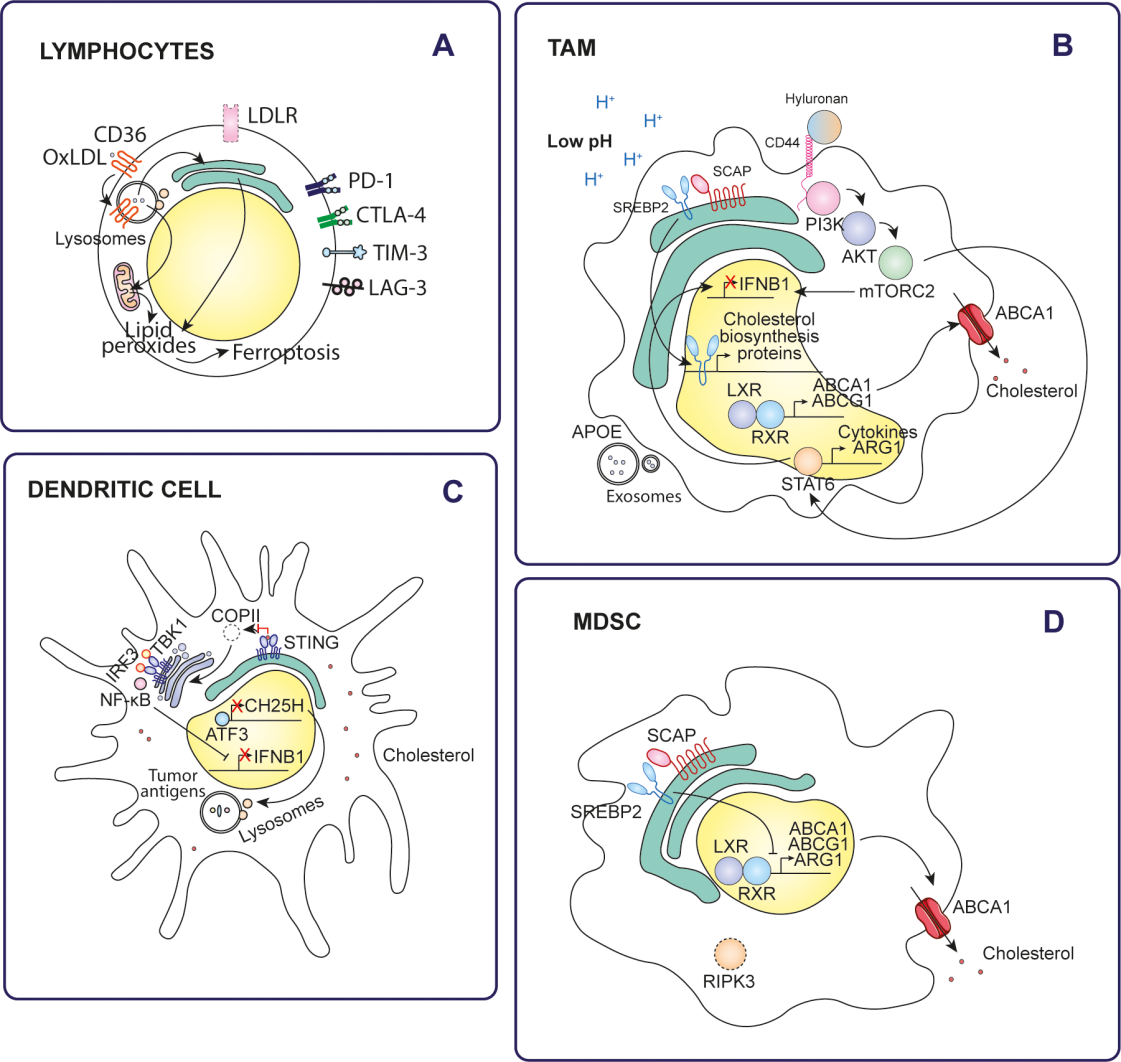
Effects of cholesterol on tumor immune cells. Cholesterol alters the antitumor immune response through various mechanisms in different types of immune cells of the tumor microenvironment. **(A)** Lymphocytes, **(B)** TAMs, **(C)** Dendritic cells, **(D)** MDSCs.

In this sense, in many cancers, the tumor microenvironment becomes enriched with cholesterol as cancer cells upregulate their cholesterol biosynthesis, enhance LDLR-mediated uptake of exogenous cholesterol, and promote cholesterol metabolism through esterification and oxidation processes (**Figure 2**) (3). Meanwhile, the surrounding cells contribute to this accumulation by increasing their cholesterol biosynthesis and raising their cholesterol efflux (**Figure 3**) (21).

Fibroblasts influence tumor cholesterol levels by secreting it and secreting growth factors that promote lipid synthesis (13), while endothelial cells facilitate cholesterol trafficking between blood vessels and tumor cells (14). Neurons release signaling molecules that impact cholesterol metabolism (15). Adipocytes release free fatty acids and cytokines, altering cholesterol levels and influencing tumor growth and immune function (16). An increased concentration of cholesterol in the tumor microenvironment can significantly affect the function of immune cells, including macrophages, lymphocytes and dendritic cells. This elevated cholesterol level causes these cells to reprogram themselves and adopt an immunosuppressive phenotype, which not only impairs their ability to mount an effective immune response, but also contributes to the production of factors that promote tumor cell growth and survival (9). Notably, these cholesterol effects have a profound impact on patient prognosis. For example, patients with hepatocellular carcinoma whose tumor microenvironment is characterized by high cholesterol metabolism often experience worse survival outcomes compared to those with lower cholesterol metabolism. This is partly explained by differences in antitumor immune activity (19). Likewise, it has been reported that SQLE expression in tumor cells correlated negatively with infiltration of key immune cells, including CD8^+^ T cells, memory B cells and activated NK cells, whereas it correlated positively with infiltration of M0- and M1-type macrophages in multiple cancer types (22). ILCs respond to oxysterols, such as 7α,25-hydroxycholesterol, in the tumor microenvironment. These oxysterols regulate ILC migration by binding to the G-protein coupled receptor 183 (GPR183), influencing their positioning and activity within the tumor (20, 23).

### 4.1 Cancer cells

**Figure 4:**
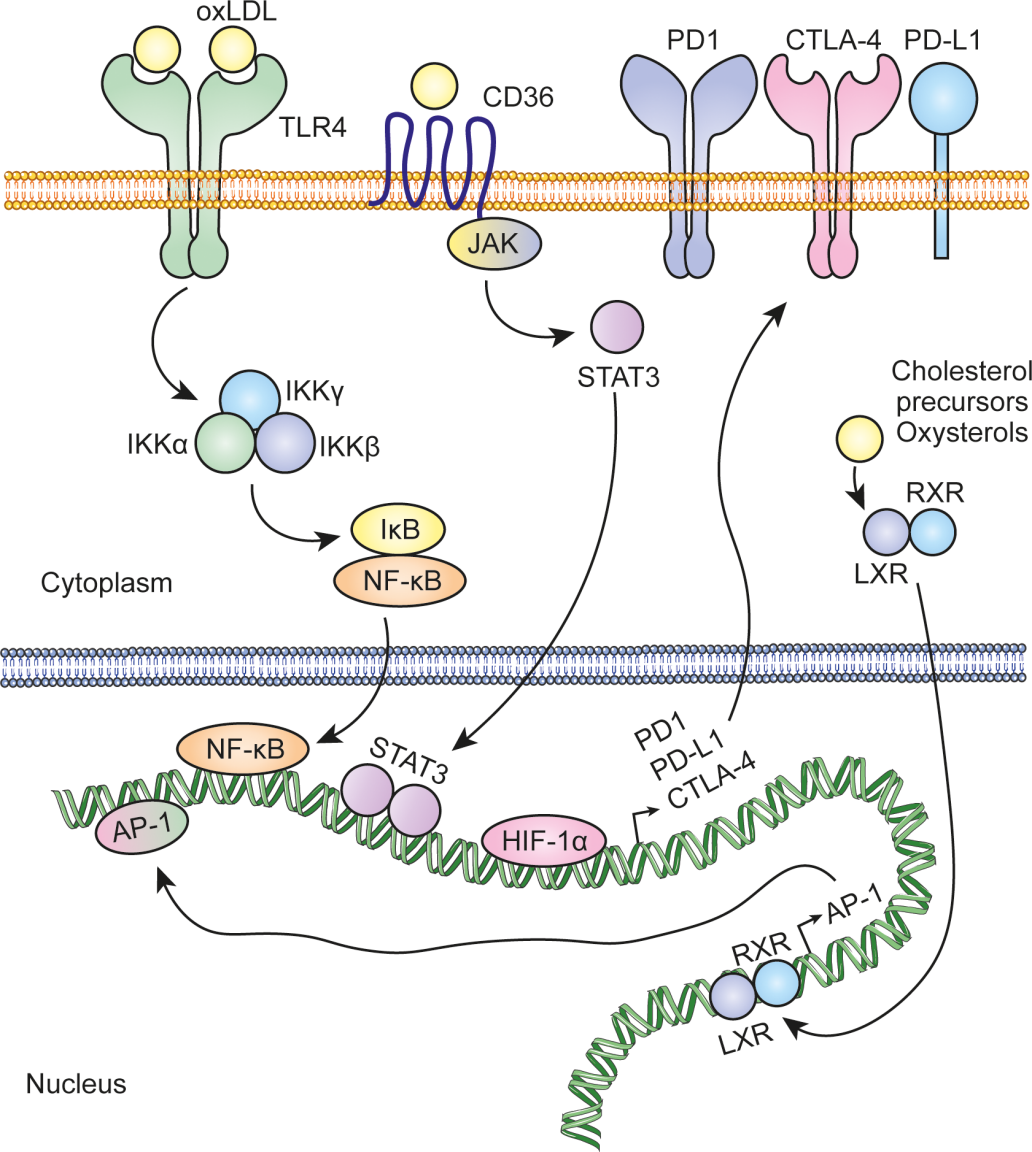
Regulation of immune checkpoint protein expression by cholesterol and its metabolites. Cholesterol and its metabolites can activate key transcription factors, including NF-kB, AP-1, and STAT3, which in turn regulate the expression of immune checkpoint proteins such as PD-L1, PD-1, and CTLA-4. In addition, hypoxia conditions in the tumor microenvironment stabilize HIF-1α, which also regulates the transcription of these proteins.

Cholesterol metabolism in cancer cells is intrinsically regulated by a dynamic interplay between oncogenes and tumor suppressors. Activation of oncogenes correlates positively with increased cholesterol metabolism, driving processes such as biosynthesis, uptake and esterification. In contrast, tumor suppressor activity acts as a brake, limiting these processes to maintain metabolic balance. Therefore, loss of tumor suppressors during tumorigenesis alters this regulation, leading to uncontrolled cholesterol metabolism (3). On the other hand, in the challenging conditions of the tumor microenvironment, characterized by hypoxia and limited nutrient availability, cells often receive extrinsic signals that affect cholesterol metabolism. Hypoxia-inducible factor-1α (HIF-1α) regulates the overexpression of the enzyme cholesterol 24-hydroxylase (CYP46A1). CYP46A1 produces 24-hydroxycholesterol (24HC), which plays a crucial role in the activation of the angiogenic switch. This process involves the recruitment and positioning of proangiogenic neutrophils near CYP46A1+ islets, promoting the formation of new blood vessels and favoring tumor progression (24). Acidification of the tumor microenvironment, driven mainly by increased cancer cell glycolysis and subsequent lactate production and secretion, plays a key role in increased cholesterol biosynthesis. In this context, low pH conditions in tumors activate SREBP2 enhancing cholesterol biosynthesis (25). Similarly, cells experience ER stress and an unfolded protein response (UPR) in an adaptive response. During ER stress, activation of X-box binding protein 1 (XBP1) plays a crucial role in the stabilization and activation of SREBP2 (26). Elevated cholesterol levels amplify nuclear factor κ-light-chain-enhancer of activated B cells (NF-κB) signaling, creating a positive feedback loop in which NF-κB further potentiates cholesterol biosynthesis and uptake (27). Moreover, elevated levels of cholesterol or its metabolites can induce the expression of immune checkpoint proteins through various mechanisms (**Figure 4**). This, in turn, enables cancer cells to suppress the antitumor activity of tumor-infiltrating leukocytes. Part of the cholesterol is encapsulated in small extracellular vesicles, which are taken up by MDSCs by macropinocytosis. Once internalized, cholesterol drives the expansion of MDSCs, enhancing their immunosuppressive capacity. This, in turn, inhibits CD8^+^ T cell activation, further suppressing the immune response against the tumor and promoting a more immunosuppressive tumor microenvironment (28).

Remarkably, different types of cancer use different mechanisms to increase cholesterol levels. Similarly, cholesterol contributes to immune suppression through a variety of pathways that vary between cancer types. The following sections will discuss these mechanisms in detail for each specific case. In addition, **Figure 2** illustrates the mechanisms of five types of cancer.

#### 4.1.1 Glioma

Gliomas account for approximately 26% of all brain tumors and 81% of malignant brain tumors. Among them, glioblastomas are the most aggressive subtype, characterized by rapid progression and limited treatment options. Prognosis remains poor, with five-year survival rates ranging from just 0.05% to 4.7% (29). Glioma tumors are characterized by significantly higher extracellular cholesterol uptake compared to healthy tissue. In this sense, glioma cells stimulate astrocytes to increase cholesterol production and ABCA1-mediated efflux to meet their demands. Notably, cholesterol is especially enriched in the mitochondrial membranes of gliomas, which reduces membrane fluidity and sensitivity to cellular stress. This adaptation increases the cells’ resistance to adverse conditions, ultimately favoring their survival and persistence (30). Notably, cholesterol depletion disrupts lipid rafts, altering membrane structure and activating disintegrin and metalloproteinase domain-containing protein 10 (ADAM10), a protease that cleaves the CD44 receptor on the cell surface. This cleavage inhibits cell migration driven by hyaluronic acid oligosaccharides or epidermal growth factor (EGF) (31).

Specifically, in glioblastoma, microglia subjected to oxidative stress shows an increased binding of nuclear receptor subfamily 4 group A member 2 (NR4A2) to the promoter region of the SQLE gene. This activation increases cholesterol biosynthesis, which contributes to immunosuppression within the tumor microenvironment. Pharmacological inhibition of the NR4A2-SQLE axis has been shown to reduce immunosuppression and improve the efficacy of ICB therapies, highlighting its potential as a therapeutic target in glioblastoma (32).

Furthermore, cholesterol in glioblastoma is redistributed and enriched in the tumor microenvironment, especially in the form of HDL-type cholesterol, due to the metabolic co-dependence between tumor cells and their environment. This cholesterol enrichment leads to cholesterol accumulation in glioblastoma infiltrating macrophages and microglia-derived tumor-associated macrophages (MN-TAMs) (33, 34). Cholesterol accumulation renders MN-TAMs deficient in phagocytosis by up-regulating phagocytosis-inhibitory receptors, such as siglec-10 and programmed cell death protein 1 (PD-1). Potentiation of cholesterol efflux by apolipoprotein A1 (ApoA1), a reverse cholesterol transporter, restores the phagocytic activity of MN-TAMs and reactivates macrophage-mediated antitumor immunity. ApoA1 facilitates lipid-related metabolic remodeling in TAMs by reducing levels of 7-ketocholesterol, a metabolite that directly inhibits tumor necrosis factor (TNF) signaling by altering mitochondrial translation (33).

#### 4.1.2 Hepatocellular carcinoma

Liver cancer is the fifth most commonly diagnosed cancer and the third leading cause of cancer-related mortality, accounting for 9.4% of all cancer deaths. The majority of primary liver cancers are hepatocellular carcinomas (HCC), accounting for 75% to 85% of cases (35). In HCC, cholesterol plays a dual role, with its levels and the involved cells being critical factors. High cholesterol levels act as tumor promoters by remodeling the hepatic immune repertoire and fostering a tumorigenic microenvironment. This process activates hepatic stellate cells to support tumor proliferation, alters membrane fluidity, and disrupts intercellular communication and immune signaling, ultimately impairing antitumoral responses. Conversely, maintaining optimal cholesterol levels can inhibit HCC development by activating NK cells to target hepatoma cells and facilitating the translocation of CD44 into lipid rafts. This reduces CD44-mediated migration and metastasis, helping to limit tumor spread (36).

In nonalcoholic fatty liver disease (NAFLD)-associated HCC, cancer cells show an elevated expression of N^6^-methyladenosine (m6A)-methyltransferase catalytic subunit (METTL3). METTL3 catalyzes m6A methylation of SCAP mRNA, enhancing its translation. This process activates SREBP2, driving production and secretion of elevated levels of cholesterol and cholesteryl esters by tumor cells. The accumulation of these lipids in the tumor microenvironment induces CD8^+^ T cell dysfunction, promoting T cell exhaustion and diminishing their cytotoxic capacity (37).

Similarly, metabolically dysfunctional steatohepatitis-associated HCC (MASH-HCC) exhibits overexpression of SQLE, leading to cholesterol accumulation in the tumor microenvironment. This dysregulated cholesterol metabolism affects immune cell function, inducing mitochondrial dysfunction in CD8^+^ T cells and MDSCs (38).

BOS complex subunit transmembrane protein 147 (TMEM147) is upregulated in hepatocellular carcinoma (HCC) cells. TMEM147 signaling increases transcription of 7-dehydrocholesterol reductase (DHCR7) through activation of signal transducer and activator of transcription 2 (STAT2). Consequently, cellular cholesterol homeostasis is altered and leads to elevated extracellular levels of 27-hydroxycholesterol (27HC). Elevated 27HC levels in hepatocellular carcinoma cells upregulate glutathione peroxidase 4 (GPX4), leading to ferroptosis resistance and promoting cancer proliferation (39). Ferroptosis is a form of regulated cell death driven by iron-dependent accumulation of lipid peroxides. This process involves the accumulation of redox-active iron, which promotes the generation of ROS through Fenton reactions. The resulting lipid peroxides induce oxidative damage to cell membranes, ultimately compromising membrane integrity and leading to cell death (40). The protective role of cholesterol is also attributed to its ability to decrease membrane fluidity and promote the formation of lipid rafts, thus regulating negatively the diffusion of lipid peroxidation substrates (41). Furthermore, secretion of 27HC by hepatocellular carcinoma cells enhances lipid metabolism in macrophages and activates peroxisome proliferator-activated receptor-γ (PPARγ) signaling, driving M2-type macrophage polarization (39). Likewise, natural killer T (NKT) cells experience decreased cytotoxicity due to lipid-induced dysfunction (42).

Transforming growth factor β1 (TGF-β1) secreted by cancer-associated fibroblasts (CAFs) activates JUN signaling in hepatocellular carcinoma cells, leading to increased semaphorin-3C (SEMA3C) expression. Released SEMA3C binds to neuropilin-1 (NRP1) and integrin β1 (ITGβ1) receptors on hepatic stellate cells (HSC), triggering NF-κB signaling. This activation stimulates interleukin-6 (IL-6) release and upregulates HMGCR expression, driving increased cholesterol synthesis. Increased cholesterol levels further activate HSCs, promoting the formation of a supportive niche within the tumor microenvironment (43).

#### 4.1.3 Colorectal cancer

Colorectal cancer is the third most commonly diagnosed cancer, accounting for approximately 9.6% of all global cancer cases. It is also the second leading cause of cancer-related deaths, responsible for 9.3% of total cancer fatalities (35). In colorectal cancer, dual specificity protein phosphatase 18 (DUSP18) dephosphorylates and stabilizes the upstream stimulatory factor 1 (USF1) transcription factor, which subsequently induces the expression of the SREBF2 gene. This regulation allows tumor cells to accumulate the cholesterol biosynthesis intermediate, lanosterol, and release it into the tumor microenvironment. Within the tumor microenvironment, lanosterol is taken up by tumor-infiltrating CD8^+^ T cells, where it suppresses the mevalonate pathway. This suppression reduces GTPase KRAS protein prenylation and function and ultimately affects CD8^+^ T cell activation. This mechanism establishes a molecular basis for immune escape of tumor cells (44).

V-type proton ATPase 116 kDa subunit a 1 (ATP6V0A1) promotes cholesterol uptake in colorectal cancer cells by facilitating endosome maturation through rabaptin-5 GDP/GTP exchange factor (RABGEF1). This process leads to the accumulation of cholesterol in the endoplasmic reticulum and increased production of 24-hydroxycholesterol (24HC). ATP6V0A1-induced 24HC activates LXR signaling, which upregulates TGF-β1. Subsequently, colorectal cancer cells release TGF-β1 into the tumor microenvironment, where it activates the mothers against decapentaplegic homolog 3 (SMAD3) pathway in memory CD8^+^ T cells, ultimately impairing their antitumor activity (45).

LSS is downregulated in tumor cells, leading to intracellular accumulation of 2,3-oxidosqualene. This metabolite specifically targets and stabilizes PD-L1, which suppresses the activity of tumor-infiltrating T lymphocytes and leads to increased recruitment and activity of polymorphonuclear myeloid-derived suppressor cells (PMN-MDSCs) and tumor-associated macrophages (TAMs) (46).

In microsatellite stable (MSS) colorectal cancer, tumor cells exhibit asynchronous disruption of the cholesterol biosynthesis pathway, leading to the accumulation of distal cholesterol precursors such as desmosterol and zymosterol. Once released, these cholesterol precursors interact with LDLR of T cells, driving their polarization towards a Th17 phenotype through activation of the transcription factor RORγt, which has immunosuppressive functions (47).

#### 4.1.4 Prostate cancer

Prostate cancer, which accounts for 7.3% of all cancers diagnosed worldwide, is the second most common cancer in men. Despite its high incidence, it ranks eighth in cancer-related mortality, accounting for 4.1% of all cancer deaths (35). In prostate adenocarcinoma, elevated stearoyl-CoA desaturase (SCD) expression in malignant cells is positively associated with increased infiltration of CD8^+^ T cells and macrophages. Furthermore, SCD expression correlates negatively with immunosuppressive molecules, such as lymphocyte activation gene 3 protein (LAG3), galectin-9 (GAL9), programmed cell death 1 ligand 1 (PD-L1) and cytotoxic T-lymphocyte protein 4 (CTLA4), whereas it shows positive correlations with immune activation molecules such as tumor necrosis factor receptor superfamily member 18 (TNFRSF18), tumor necrosis factor ligand superfamily member 4 (TNFSF4) and CD47. SCD promotes the production of monounsaturated fatty acids, which serve as substrates for cholesterol esterification. This activity facilitates the storage of cholesterol in lipid droplets, organizes lipid rafts within cell membranes and regulates free cholesterol levels (48).

Reducing cholesterol levels in castration-resistant prostate cancer tissues resulted in decreased immunosuppressive macrophage infiltration. In addition, it significantly reduced transcriptional programs involved in steroid and bile acid synthesis, which in turn reduced androgen synthesis and inhibited androgen receptor (AR) nuclear localization. The mechanism also involves prevention of LXR activation. All these facts improved the survival outcomes of prostate cancer patients during androgen deprivation therapy (49).

#### 4.1.5 Breast cancer

Breast cancer is the second most common cancer overall and the most common cancer in women. It is also the leading cause of cancer-related deaths among women (35). Breast cancer cells exposed to high cholesterol levels promote increased macrophage infiltration, with a shift toward an M2-type, along with increased angiogenesis and endothelial branching. Then, these conditions drive the development of a CAF phenotype, which contributes to tumor progression. These effects may be attributed to the activation by cholesterol of the estrogen-related α-receptor (ERRα) pathway in macrophages and the release of cytokines by cancer cells, such as eotaxin-1, interleukin-1β (IL-1β), interleukin-3 (IL-3) and IL-6 (50).

Breast cancer cells overexpressing tetraspanin-6 (TSPAN6) promote B lymphocyte infiltration of tumors by driving the accumulation of oxysterol ligands, such as 25-hydroxycholesterol (25HC) and 27HC, within cell-derived extracellular vesicles. These oxysterols activate the LXR signaling pathway in B lymphocytes, triggering an LXR-dependent transcriptional network that potentiates cholesterol efflux (51).

Cholesterol sulfate, synthesized by sulfotransferase 2B1 (SULT2B1) in tumor cells, is secreted to suppress dedicator of cytokinesis protein 2 (DOCK2) enzymatic activity in T cells, leading to CD8^+^ T cell exhaustion (52). Tumors generated from breast cancer cells overexpressing SULT2B1 showed significant changes in immune cell populations and cytokine expression. Specifically, there was a reduction in CD11b^+^Ly6G^+^ neutrophils, along with an increase in F4/80-CD11b^+^CD11c^+^ dendritic cells and γδ T cells, which play key roles in the immune response. In addition, these tumors showed elevated levels of anti-inflammatory cytokines, such as IL-10, IL-4 and IL-13 (53).

#### 4.1.6 Lung cancer

Lung cancer is the most commonly diagnosed cancer worldwide and the leading cause of cancer death (35). In epidermal growth factor receptor (EGFR)-wild-type non-small cell lung cancer (NSCLC), stratification based on glycolytic and cholesterol-related gene expression levels highlights a subgroup of tumors with significant enrichment of cholesterol gene signatures. These tumors are associated with the worst prognosis, particularly in immuno-cold NSCLC (54).

Overexpression of disintegrin and metalloproteinase domain-containing protein 9 (ADAM9) in lung cancer reduced LDLR protein stability by promoting its proprotein convertase subtilisin/kexin type 9 (PCSK9)-mediated degradation, leading to decreased cholesterol uptake and triggering cholesterol biosynthesis. This, in turn, increased IL-6 expression and secretion through activation of signal transducer and activator of transcription 3 (STAT3) signaling. Cancer cell-derived IL-6 inhibited interleukin-12 subunit β (IL-12β) secretion and antigen presentation by dendritic cells, rendering them unable to activate cytotoxic CD8^+^ T cells (55).

#### 4.1.7 Gastric cancer

Two distinct subtypes of cholesterol metabolism have been identified in gastric cancer. The first subtype is characterized by increased cholesterol biosynthesis, whereas the second subtype shows impaired cholesterol transport. In particular, this second subtype is associated with more unfavorable clinical features, an enrichment in malignant signaling pathways and a tumor-friendly immune microenvironment compared to the first. In this regard, there was a higher abundance of naïve B lymphocytes, γδ T lymphocytes, M2-type macrophages, resting dendritic cells, resting mast cells and eosinophils compared to the first subtype. In contrast, it had lower levels of plasma cells, follicular helper T lymphocytes, activated NK cells, M0-type macrophages, activated dendritic cells, activated mast cells and neutrophils (56).

#### 4.1.8 Esophageal cancer

Esophageal cancer is the eleventh most commonly diagnosed cancer globally and the seventh leading cause of cancer-related deaths worldwide (35). In metastatic esophageal squamous cell carcinoma (ESCC), tumor cells upregulate anoctamin-1 (ANO1), which drives both cell-intrinsic and cell-extrinsic mechanisms to alter cholesterol metabolism and stimulate fibroblast activity. ANO1 plays a key role in inactivating the LXR pathway, leading to increased intracellular cholesterol accumulation. This occurs through the interaction of ANO1 with the transcription factor JUN, which represses mitochondrial sterol 26-hydroxylase (CYP27A1) expression and induce interleukin-1β (IL1β) secretion (57).

#### 4.1.9 Nasopharyngeal cancer

Nasopharyngeal cancer is the 23rd most diagnosed cancer in the world and ranks 21st in terms of cancer-related mortality (35). Nasopharyngeal carcinoma cells promote the development and suppressive activity of Tregs through the interaction of CD70 and CD27. CD70 on nasopharyngeal carcinoma cells binds to CD27 on naïve CD4^+^ T cells, driving differentiation to Tregs and their activation. This interaction leads to intracellular accumulation of cholesterol in Tregs, along with increased fatty acid and cholesterol metabolism in mitochondria. Subsequently, activated Treg cells release soluble CD27, interleukin-10 (IL-10), TGF-β and adenosine to limit the effective antitumor response of CD8^+^ cytotoxic T cells. Inhibition of CD70-CD27 signaling causes lipid efflux and promotes their elimination, reversing the phenomenon and enhancing antitumor immunity (58).

#### 4.1.10 Kidney cancer

Kidney cancer is the 14th most commonly diagnosed cancer in the world and the 16th leading cause of cancer death worldwide (35). Elevated cholesterol synthesis in Wilms tumor is associated with poor prognosis. It negatively affects the tumor microenvironment by reducing cytolytic activity, impairing dendritic cell (DC) function, and weakening the major histocompatibility complex (MHC I) signature, all of which contribute to decreased antitumor immune response (59).

### 4.2 Immune cells

Tissue-resident immune cells and tumor-associated immune cells exhibit significant differences in cholesterol metabolism due to their distinct microenvironments (60). In healthy tissues, immune cells tightly regulate cholesterol homeostasis through controlled LDL uptake via the LDLR, efficient biosynthesis via the mevalonate pathway, and balanced cholesterol efflux, ensuring proper immune function and activation. They rely primarily on oxidative phosphorylation and glycolysis for energy, and cholesterol metabolism contributes to membrane dynamics and intracellular signaling (61). In contrast, tumor-associated immune cells, such as TAMs and MDSCs, often show increased cholesterol biosynthesis and uptake through lipoprotein and scavenger receptors (e.g., CD36), along with impaired cholesterol efflux, leading to cholesterol accumulation. These cells undergo metabolic reprogramming, switching to one with enhanced lipid metabolism, which increases their tumor-promoting activity (61).

Cholesterol, as a key component of cell surface lipid rafts, plays a crucial role in immune signaling. For example, in tyrosine kinase signaling, ligand activation recruits adaptors, scaffolds and enzymes on the cytoplasmic side of the plasma membrane. Lipid rafts facilitate this process by pooling activated receptors and protecting the signaling complex from non-active enzymes, such as membrane phosphatases, that might otherwise disrupt the signaling cascade. Consequently, alterations in cholesterol levels in immune cells may affect intercellular communication, receptor-mediated immune activation and downstream signaling pathways, among other processes (62).

#### 4.2.1 Lymphocytes

In the cholesterol-rich tumor microenvironment, CD8^+^ T cells uptake and accumulate cholesterol after migrating to the tumor. This accumulation of cholesterol significantly reduces their antitumor activity. Mechanistically, elevated cholesterol levels in these tumor-infiltrating CD8^+^ T cells trigger ER stress and activation of XBP1 signaling pathway which, in turn, leads to the expression of immune checkpoint molecules such as PD-1, natural killer cell receptor 2B4, metalloproteinase inhibitor 3 (TIM3) and lymphocyte activation gene 3 protein (LAG3) (63). Indeed, T cells infiltrating tumors that respond to immune checkpoint inhibitors (ICIs) exhibit increased cholesterol uptake compared to those from non-responsive tumors (64).

In hepatocellular carcinoma, tumor-secreted fibroblast growth factor 21 (FGF21) drives AKT activation in T cells, which stimulates mammalian target of rapamycin target complex 1 (mTORC1). In turn, mTORC1 activates SREBP2 and consequently increases transcription of genes essential for cholesterol production. The resulting accumulation of cholesterol induces T-cell exhaustion (65).

Spatial analysis of immune cells in triple-negative breast cancer identified distinct meta-signatures based on CD8^+^ T-cell localization. Tumors with CD8^+^ T cells predominantly accumulating in the stromal region were characterized by an up-regulation of cholesterol biosynthesis. These tumors also showed the presence of interleukin-17 (IL-17) producing cells, Tregs and neutrophils, along with a mutual exclusion of type 1 interferon (IFN) responses. In addition, these tumors were enriched with immune modulators such as programmed cell death 1 ligand 1 (PD-L1) and indoleamine 2,3-dioxygenase 1 (IDO1), further underscoring the immunosuppressive nature of the stromal environment (66).

Possibly as a strategy to reduce cholesterol intake, LDLR expression is significantly lower in total CD8^+^ colorectal tumor infiltrating lymphocytes (TILs) compared to active CD8^+^ TILs. However, this reduced LDLR expression compromises their ability to produce cytokines, release granules and undergo clonal expansion upon stimulation. LDLR-mediated LDL uptake plays a critical role in priming and expansion of naïve CD8^+^ T cells. In addition, LDLR interacts with the T cell receptor (TCR) and modulates TCR signaling, which is essential for CD8^+^ T cell activation and functional response (67).

Furthermore, platelet glycoprotein 4 (CD36) facilitated the uptake of oxidized low-density lipoproteins (OxLDLs) into T cells (68). Increased CD36 expression on tumor-infiltrating CD8^+^ T cells, compared to normal tissue T cells, is driven by high cholesterol levels in the tumor microenvironment and is associated with tumor progression and poor survival. OxLDL uptake triggers lipid peroxidation and ferroptosis, which reduces cytotoxic cytokine production and decreases antitumor activity. Blockade of CD36 or inhibition of ferroptosis in CD8^+^ T cells effectively restore their antitumor function. In particular, these interventions demonstrate even greater antitumor efficacy when combined with anti-PD-1 immunotherapy (69).

One study reported that lymphocytes with antitumor activity, derived from various tumor models, exhibited an enrichment of the APOA1 protein (70). APOA1 facilitates ABCA1-mediated cholesterol efflux, reducing intracellular cholesterol levels in these cells. By reducing cholesterol, APOA1 prevents CD8^+^ T cell exhaustion, thereby enhancing their antitumor activity (71). APOA1 shows a positive correlation with antitumor immune cells, such as NK cells and CD8^+^ T cells, whereas it shows a negative association with immunosuppressive immune cells, including M2-type macrophages.

Recent studies have revealed that cancer cells can transfer damaged mitochondria to TILs, profoundly impairing their antitumor functions (72). In this context, the accumulation of oxysterols in these lymphocytes may be further intensified by increased ROS production, a consequence of mitochondrial dysfunction. In addition, excess cholesterol would disrupt the oxidative phosphorylation process in the remaining functional mitochondria, further impairing ATP production and decreasing the energy supply necessary for an effective immune response.

#### 4.2.2 Dendritic cells

Increasing cholesterol levels, either through a cholesterol-rich diet, apolipoprotein E (APOE) ablation, or inhibition of cholesterol efflux by LXR suppression, impairs dendritic cell migration (73, 74). Similarly, 22R-hydroxycholesterol (22R-HC)-treated monocyte-derived dendritic cells show reduced migration, accompanied by decreased expression of the chemokine receptor CCR7 and impaired T-cell priming (75).

Cholesterol inhibits stimulator of interferon genes (STING)-dependent antitumor activity by disrupting STING signaling in monocyte-derived dendritic cells. Cholesterol directly binds to STING at its cholesterol-recognition motifs (CRAC), disrupting its activation. This prevents the proper formation of the coat protein complex II (COPII), which is necessary for STING transport from the ER to the Golgi. As a result, STING is trapped in the ER, preventing it from recruiting TBK1 and activating interferon regulatory factor 3 (IRF3). This alteration inhibits the downstream signaling cascade necessary for the expression of type I interferons (IFN) and other proinflammatory cytokines, which are essential for mounting an effective antitumor immune response (76).

Furthermore, cholesterol metabolism affects antigen presentation by dendritic cells. In lung cancer, intratumoral dendritic cells show reduced expression of cholesterol 25-hydroxylase (CH25H) due to activation of activating transcription factor-3 (ATF3) by tumor-derived factors. Downregulation of CH25H alters endosomal dynamics by promoting membrane fusion between endophagosomes and lysosomes. This accelerates lysosomal degradation of tumor antigens, thereby limiting their cross-presentation to T cells. As a result, dendritic cells in the tumor microenvironment become less effective in stimulating antitumor immunity (77).

#### 4.2.3 Macrophages

Like dendritic cells, macrophages are also affected by cholesterol metabolism within the tumor microenvironment. Low pH conditions in tumors activate SREBP2 and LXR in macrophages, enhancing their biosynthesis and efflux of cholesterol to meet the metabolic demands of tumor cells (78). In this regard, higher SREBP2-mediated cholesterol biosynthesis and ABCG1-mediated cholesterol export were observed in pancreatic cancer TAMs compared to healthy human pancreatic macrophages (79, 80). In addition, autocrine cytokines secreted by tumor-induced monocytes further amplify the expression of the transcription factors SREBP2 and LXR, enhancing their activation and downstream signaling pathways (81).

A subset of tumor-associated macrophages, known as lipid-laden macrophages, plays a critical role in aggressive mesenchymal glioblastoma. These macrophages are distinguished by their large size and the accumulation of abundant perilipin-2 positive (PLIN2+) lipid droplets, reflecting elevated cholesterol levels. Through an LXR/ABCA1-dependent pathway, lipid-laden macrophages transfer myelin-derived lipids directly to cancer cells, meeting the elevated metabolic demands of glioblastoma cells and protecting them from lipotoxicity. These effects favor tumor survival, contribute to resistance against therapy, and are associated with poor prognosis in patients (82).

Ovarian cancer cells drive tumor-associated macrophage (TAM) development and enhance cholesterol efflux by inducing tumor endothelium to secrete C-X-C motif chemokine 2 (CXCL2). CXCL2 activates the NOTCH/recombining binding protein suppressor of hairless (RBPJ) signaling pathway, leading to upregulation of CD44 on monocytes. In turn, high molecular weight (>100 kDa) tumor-derived hyaluronan (HA) in the tumor microenvironment binds to CD44 and activates the phosphatidylinositol 3-kinase (PI3K)/AKT/mammalian target of rapamycin complex 1 (mTORC2) signaling pathway. This activation culminates in the expression of STAT6 target genes, suppressing interferon-γ (IFNγ) signaling. As a result, macrophages reprogram into an immunosuppressive TAM phenotype, fostering a tumor-promoting microenvironment (21, 83).

In contrast to dendritic cells, M2-type macrophages with high levels of CH25H are enriched in immunosuppressive tumor microenvironments and are associated with reduced survival rates in several types of cancer. CH25H expression in these cells is induced by factors such as lactic acid, acidic pH, interleukin-4 (IL-4) or interleukin-13 (IL-13), which act through the signal transducer and transcription activator 6 (STAT6). This leads to the accumulation of 25HC. In the lysosome, 25HC competes with cholesterol for binding to the lysosomal cholesterol signaling protein (LYCHOS) receptor, which inhibits mTORC1 kinase. This inhibition activates AMPKα, driving metabolic reprogramming of cells. In a feedback mechanism, AMPKα phosphorylates STAT6 at S564, amplifying STAT6 activation and further potentiating its signaling activity. Simultaneously, STAT6 drives the expression of arginase-1 (ARG1), an enzyme that depletes arginine from the tumor microenvironment. This reduction in arginine availability inhibits T cell proliferation. Targeting 25HC, alone or in combination with anti-PD-1 immunotherapy, has been shown to improve the efficacy of immunotherapy by reprogramming the tumor microenvironment and reducing immunosuppression (84).

Apolipoprotein E (APOE) is highly expressed in TAMs infiltrating pancreatic ductal adenocarcinoma (PDAC) compared to macrophages in adjacent tissues. This elevated expression is strongly associated with an immunosuppressive phenotype. Through the LDLR and NF-κB signaling pathways, APOE induces tumor cells and fibroblasts to produce the immunosuppressive C-X-C motif chemokine 1 (CXCL1) and C-X-C motif chemokine 5 (CXCL5). These chemokines recruit immature myeloid cells into the tumor microenvironment, which in turn inhibits CD8^+^ T-cell infiltration (85).

Increased macrophage colony-stimulating factor 1 receptor (CSF1R) expression in TAMs within colorectal cancer drives their reprogramming towards the immunosuppressive phenotype. This process is mediated by activation of the PI3K/AKT/mammalian target of rapamycin complex 1 (mTORC1) signaling pathway, which enhances cholesterol biosynthesis. Elevated cholesterol levels contribute to the creation of an immunosuppressive tumor microenvironment, which hinders CD8^+^ T-cell infiltration and correlates with poor patient survival outcomes (86).

#### 4.2.4 Myeloid-derived suppressor cells

Fibroleukin (FGL2) drives the differentiation and immunosuppressive activity of MDSCs through FGL2-low affinity immunoglobulin γ Fc region receptor IIB (FcγRIIB) signaling. This pathway induces the generation of reactive oxygen species (ROS), leading to lipid peroxidation and ER stress. Blockade of FGL2 disrupts this pathway, reducing MDSC-mediated immunosuppression and significantly improving the efficacy of immune checkpoint inhibitors, such as anti-PD-1 therapy (87).

Receptor-interacting serine/threonine-protein kinase 3 (RIPK3) is deficient in tumor-infiltrating MDSCs, resulting in suppression of cholesterol synthesis. Normally, RIPK3 promotes cholesterol synthesis and accumulation in MDSCs through the AKT-mTORC1 signaling pathway, which activates the downstream SREBP2-HMGCR axis. Reduced cholesterol levels potentiate the immunosuppressive activity of MDSCs. Mechanistically, cholesterol depletion induces nuclear accumulation of liver X receptor β (LXRβ), which forms a heterodimer with retinoic acid receptor RXRα (RXR). This heterodimer binds to the arginase-1 (ARG1) promoter. ARG1 causes immunosuppression by depleting L-arginine, which affects T-cell proliferation and activation (88).

## 5 Dietary cholesterol

The role of dietary cholesterol in the immune components of the tumor microenvironment remains poorly understood, and the available research is limited to draw definitive conclusions. Nevertheless, this section aims to draw attention to this issue and to encourage the scientific community to investigate further. Although the evidence is still incipient, some studies suggest that high cholesterol intake may have an immunosuppressive effect. It is important to consider that prolonged high cholesterol diet (HCD) can lead to chronic systemic inflammation, resulting in an increased accumulation of dysfunctional and senescent immune cells (89). This may reduce the number of functional leukocytes available to infiltrate tumors and perform their antitumor activity.

Additionally, elevated levels of circulating cytokines could further impair immune cell function, weakening the overall immune response against cancer.

In colorectal cancer, a high cholesterol diet (HCD) favors the infiltration of macrophages, which secrete the chemokine C-C motif ligand 5 (CCL5). This secretion triggers the binding of NF-κB p65 and STAT3 to the promoter region of the COP9 signalosomal complex subunit 5 (CSN5) gene, causing an increase in its expression. Elevated levels of CSN5 subsequently regulate programmed cell death 1 ligand 1 (PD-L1) stability in cancer cells by modulating its deubiquitination (90).

Dyslipidemia induced in mice by a diet high in fat and cholesterol significantly reduced dendritic cell activation in mice tumor microenvironment. This, in turn, profoundly affected T helper cell responses, leading to an impairment of Th1 responses and an increase in immunosuppressive Th2 responses (91). Furthermore, it has also been reported that a cholesterol-rich diet can weaken the effects of hydroxypropyl-β-cyclodextrin (HP-β-CD), a lipid raft disruptor, in reducing the expression of immune checkpoint molecules in breast cancer mice models (92).

While exogenous cholesterol alone had minimal direct effects on macrophages, it synergized with tumor-derived factors to further increase ABCA1 expression, promoting macrophages with stronger immunosuppressive characteristics. Tumors with elevated ABCA1+ macrophage numbers were associated with lower CD8^+^ T-cell infiltration and worse clinical outcomes in patients with hepatocellular carcinoma (81).

Patients with high serum cholesterol levels showed shorter survival times and lower response rates to anti-PD-1 therapy (81). One study reported that patients with low serum HDL levels had a reduced density of secondary and tertiary lymphoid structures (TLS), but a higher density of NK cells in the extra-TLS zone. TLS are organized clusters of immune cells that form near tumors and play a crucial role in coordinating antitumor immunity. Meanwhile, compared to patients with normal LDL levels, those with low LDL levels exhibited a higher ratio of PD-1+ T follicular helper cells to CD20^+^ B cells within TLS, as well as a higher ratio of PD-1^+^ T cells to CD8^+^ T cells. Additionally, they showed an increased density of PD-1^+^ T cells in the extra-TLS zone (93).

Omega-3 fatty acids (ω-3 FA) from fish oil exert effects through free fatty acid receptor 4 (FFAR4) on functional bone marrow-derived immune cells that migrate to the tumor microenvironment. This action reduces the infiltration of CD206^+^ (M2) macrophages into the tumor and down-regulates the expression of the cholesterol transporter genes ABCA1, ABCG1 and ATP-binding cassette sub-family A member 6 (ABCA6), as well as the cholesterol synthesis-related genes mitochondrial acetyl-CoA acetyltransferase (ACAT1) and cytosolic acetyl-CoA acetyltransferase (ACAT2) in these cells (94).

Despite all these findings, it remains unclear whether dietary cholesterol could be classified as a risk factor for promoting a more immunosuppressive tumor microenvironment. Therefore, further research is needed to explore the systemic effects of cholesterol on both the tumor microenvironment and the immune system

## 6 Targeting cholesterol metabolism

Cholesterol in the tumor microenvironment can influence cancer treatment through several mechanisms, as it plays a role in the efficacy of chemotherapy, radiotherapy and immunotherapy (**Table 1**). Cholesterol depletion in tumor cells can weaken the intratumoral physical barrier by disrupting the mechanical signaling between tumor cells and the extracellular matrix, mediated by lipid rafts. This process not only destabilizes the structural integrity of the tumor, but also reprograms TAMs from the immunosuppressive M2-type to the proinflammatory M1-type. Additionally, these changes improve the penetration of therapeutic drugs into the tumor microenvironment (98). Several studies have shown that strategies targeting various points of cholesterol metabolism not only affect tumor cells, but also influence the tumor immune microenvironment (**Table 2**). These effects contribute to therapeutic benefits, highlighting the potential of this strategy to improve treatment outcomes.

**Table 1:** Immunotherapy effects of elevated cholesterol levels on the tumor microenvironment.

Affected immunotherapy	Cholesterol Effect	Reference
Immune checkpoint inhibitors (Anti-PD-1/PD-L1 therapies) (e.g., Nivolumab, Pembrolizumab)	Increases PD-L1 expression in tumor cells	(95)
Adoptive cell transfer therapies (e.g., CAR-T cells, TIL therapy)	Induces CD8+ T cell dysfunction	(63)
Cancer vaccines and dendritic dell-Based therapies	Reduces dendritic cell activation and antigen presentation	(96)
Immune checkpoint inhibitors (especially CTLA-4 inhibitors such as Ipilimumab)	Promotes Treg expansion	(97)
Macrophage-targeted Therapies Checkpoint Inhibitors (e.g., CSF-1R inhibitors)	Supports M2-type macrophage polarization	(21)

**Table 2:** Therapies targeting cholesterol metabolism and their effects on the immune tumor microenvironment.

	Drug	Target	Dosage	Effects on the immune tumor microenvironment	References
Targeting cholesterol biosynthesis	Statins	HMGCR	µM doses	Suppresses PD-L1 expression and thus activates the antitumor response.	(54, 99)
	Fatostatin	SREBPs	mg/kg	Decreases Tregs and alleviates CD8^+^ T cell exhaustion	(100)
	Terbinafine	SQLE	µg/mL	Reduced Tregs in tumors and increase cytotoxic T infiltration.	(101)
Targeting cholesterol esterification	Avasimibe	SOAT1	µM doses	Improves the formation of immune synapses.	(102)
Targeting LXR signalling	RGX-104	LXR	mg/kg	Causes MDSC depletion and increased T cell activation	(103)
Targeting cholesterol transport	Anti-PCSK9 antibodies	PCSK9	µg	Increases the levels of MHC I and MHC II in cells and, therefore, the antitumor activity of lymphocytes.	(104, 105)
Targeting lipid rafts	HP-β-CD	Lipid rafts	mM doses	Enhanced T cell infiltration and alleviated CD8^+^ T cell exhaustion	(92)
Targeting bile acids	Cholestyramine	Cholic acid Deoxycholic acid	8 mg/day	Reverses the polarization of the TAMs to M2 phenotype.	(106)

Lovastatin, a cholesterol synthesis inhibitor, has shown efficacy in reducing the proliferation of NSCLC cells both *in vitro* (10 µM) and *in vivo* (40 mg/kg/day for 20 days). In particular, it can reprogram the tumor microenvironment, converting it from a noninflamed “cold tumor” to an inflamed “hot tumor”. This transformation significantly increases tumor responsiveness to ICB therapies, such as anti-PD-1 treatment. In addition, lovastatin transcriptionally suppresses programmed cell death 1 ligand 1 (PD-L1) expression and induces ferroptosis, further underscoring its therapeutic potential in NSCLC (54).

Another statin, simvastatin (in micromolar doses) also suppresses PD-L1 expression and enhances antitumor immunity in colorectal cancer by down-regulating long non-coding RNA (lncRNA) small nucleolar RNA host gene 29 (SNHG29). SNHG29 interacts with Yes-associated protein 1 (YAP1), preventing its phosphorylation and ubiquitination-mediated degradation, thereby stabilizing the protein. Stabilized YAP1 binds to the PD-L1 promoter, promoting its transcription. By inhibiting SNHG29, simvastatin reduces YAP stability, leading to negative transcriptional regulation of PD-L1 and potentiating the antitumor immune response (99).

In a mouse model of oral squamous cell carcinoma, a nanoplatform consisting of simvastatin-loaded e6-polyethylene glycol chlorin (Ce6-PEG) and a target antibody (anti-LDLR at a dose of 1.0 mg/kg) was shown to induce tumor cell toxicity, regulate cholesterol levels, and stimulate the immune system. Interestingly, the treatment enhanced tumor infiltration of memory T cells and increased cytotoxic activity (107).

Inhibition of cholesterol synthesis through an alternative target, such as SQLE, by administration of terbinafine from a nanoplatform reduced cholesterol levels in the tumor microenvironment and suppressed tumor cell proliferation. This approach enhanced intratumoral infiltration and immune activation in oral squamous cell carcinoma by promoting the production of damage-associated molecular patterns for photoimmunotherapy. As a result, the number of Tregs in tumor tissues were reduced and the conversion of CD4+ T cells to Tregs was markedly inhibited. In contrast, the treatment increased the percentage of cytotoxic CD8+ T cells, effectively inducing antitumor immune responses (101).

Inhibition of cholesterol esterification, either by genetic ablation or by pharmacological inhibition of sterol O-acyltransferase 1 (SOAT1) (with avasimibe in micromolar doses), enhances the effector functions and proliferation of CD8^+^ T cells without affecting CD4^+^ T cells. In contrast, blocking cholesterol biosynthesis with lovastatin or cholesterol transport with U18666 has no such effect. This increased functionality is attributed to increased cholesterol in the plasma membrane of CD8^+^ T cells, which favors increased T-cell receptor (TCR) clustering and signaling. In addition, elevated membrane cholesterol levels facilitate the formation of a more efficient immune synapse, further amplifying the antitumor activity of CD8^+^ T cells (102).

A tumor-penetrating nanovesicle sensitive to matrix metalloproteinase-2 (MMP-2) was designed to target cholesterol metabolism and enhance photodynamic cancer immunotherapy. These nanovesicles accumulate inside tumors and release internalizing arginylglycylaspartic acid peptides that facilitate deeper tissue penetration. At the same time, the nanovesicles release avasimibe in both CD8^+^ T lymphocytes and tumor cells. This dual action restores T-cell functionality, reinvigorating their antitumor response, while suppressing tumor cell migration, thus amplifying the therapeutic effects against cancer (108).

Fatostatin (7.5-30 mg/kg), an inhibitor of SREBP activation, reduces intracellular cholesterol accumulation and mitigates ER stress. This dual action leads to a decrease in Tregs and alleviates CD8^+^ T cell exhaustion within the tumor microenvironment, ultimately improving antitumor immunity (100).

Clinically approved PCSK9 neutralizing antibodies (100 µg intraperitoneally) have been shown to act synergistically with anti-PD1 therapy in suppressing tumor growth in mouse cancer models. PCSK9 plays a key role in enhancing LDLR degradation through a clathrin and low density lipoprotein receptor adapter protein 1 (LDLRAP1)-mediated pathway. Mechanistically, PCSK9 can interfere with the recycling of major histocompatibility complex (MHC) class I and II molecules to the cell surface promoting their relocation to lysosomes and subsequent. Therefore, inhibition of PCSK9 enhances the lymphocyte antitumor activity (104, 105).

Associated with the above, arenobufagin (ARBU), a bufanolide steroid secreted by the toad *Bufo arenarum*, induces macrophage polarization toward an M1-type in a mouse model of hepatocellular carcinoma (HCC). ARBU (at mg/mL doses) reduces PCSK9 protein expression and potentiates LDLR signaling, which reduces cholesterol availability and promotes tumor cell apoptosis (109).

Therapeutic activation of LXR by the agonist RGXX-104 has been found to reduce MDSCs and enhance the response of cytotoxic T lymphocytes in both mice and patients. This effect is due to increased expression of APOE, which binds to the LRP8 receptors of MDSCs and triggers their apoptosis. In this way, it amplifies the efficacy of several immune therapies (103).

2-hydroxypropyl-beta-cyclodextrin (HP-β-CD) reduced cholesterol levels in triple-negative breast cancer tumors by enhancing ABCA1- and ABCG1-mediated reverse cholesterol transport. This process facilitated enhanced T cell infiltration into the tumor microenvironment and alleviated CD8^+^ T cell depletion by reducing expression of immune checkpoint molecules (92).

In murine models of SIRT5-deficient hepatocellular carcinoma, in which cholesterol levels are elevated due to the inability to convert cholesterol to bile acids, treatment with cholestyramine, a bile acid sequestrant and FDA-approved drug for hyperlipidemia, effectively inhibited the promotion of M2-type polarized TAMs (106, 110).

The combination of an anti-PD-1 antibody with lumacaftor (in micromolar doses), an FDA-approved small molecule inhibitor of DUSP18, has been shown to synergistically enhance antitumor immunity. DUSP18 is part of the signaling that triggers USF1-mediated SREBF2 expression (44).

Metformin indirectly influences cholesterol metabolism in immune cells. Metformin inhibits myeloid-derived suppressor cells and M2-type macrophages in the tumor microenvironment of colon cancer through the activation of AMPK, which subsequently suppresses mTOR signaling. This cascade results in the downregulation of the mevalonate pathway, contributing to its antitumor effects (111).

Likewise, decitabine, a DNA methylation inhibitor, indirectly suppresses premetastatic niche formation in colorectal cancer by increasing the expression of ATP-binding cassette subfamily A member 9 (ABCA9). This increases cholesterol efflux from tumor cells and promotes macrophage-dependent T-cell activation, thereby enhancing the antitumoral response (112).

Pharmacological inhibition or genetic deletion of METTL3, in combination with anti-PD-1 therapy, synergistically restores CD8^+^ T-cell function. METTL3 catalyzes m6A methylation of SCAP mRNA, enhancing its translation. This combined approach revitalizes cytotoxic T cells, enhancing their ability to mount an effective antitumor response and promoting tumor regression (37).

The ClinicalTrials.gov database (revised February 2025) (113, 114) highlights that, although numerous studies examine the impact of cholesterol-lowering drugs on cancer, only a limited number of clinical trials focus specifically on their effects on the tumor immune microenvironment. Among those that do, statins - in particular simvastatin, followed by atorvastatin - are the most studied. Other drugs under investigation include PCSK9 inhibitors, cholesterol esterification inhibitors such as ezetimibe, and bile acid sequestrants such as cholestyramine. In particular, one study (NCT06437574) is evaluating whether intensive cholesterol reduction with oral simvastatin and ezetimibe enhances CD8^+^ memory T cells and promotes CD8^+^ T cell infiltration into prostate tissue. Another trial (NCT05586360) is exploring whether oral simvastatin can mitigate YAP-mediated Treg dysfunction in prostate cancer, shedding light on potential mechanisms. Finally, one study (NCT05553834) is testing a therapeutic combination of alirocumab, a PCSK9 inhibitor, with cemiplimab, a PD-1 checkpoint inhibitor, to determine whether it can improve the characteristics of the NCSLC immune microenvironment and thus the overall efficacy of treatment.

## 7 Conclusions and future perspectives

In many types of cancer, the tumor microenvironment is markedly enriched in cholesterol due to multiple mechanisms. Cancer cells not only increase their own cholesterol production, but also uptake additional cholesterol from their microenvironment. This accumulation of cholesterol plays several crucial roles in cancer progression. It provides essential metabolites that act as structural components of cell membranes, sources of energy for metabolic demands and mediators of intracellular signaling pathways. Excess cholesterol is usually stored in the form of cholesterol esters, while oxidized derivatives, such as oxysterols, are secreted to exert immunosuppressive effects. These oxysterols inhibit the antitumor activity of infiltrating immune cells and protect cancer cells from lipid peroxidation, a process that might otherwise trigger their apoptosis.

Regarding immune cells, TAMs synthesize and release excess cholesterol to meet the metabolic needs of tumor cells. MDSCs also release cholesterol and consume other metabolites, such as L-arginine, which are essential for the proliferation and activation of T cells and other immune cells. In dendritic cells, increased cholesterol levels impair tumor antigen presentation, while in lymphocytes, excess cholesterol favors the development of a Treg phenotype and prevents the release of antitumor cytokines, further weakening the immune response against tumors.

All of these factors support the idea of targeting cholesterol metabolism as a potential strategy for cancer treatment. By disrupting the supply of essential cholesterol to cancer cells, this approach could not only inhibit tumor growth, but also enhance antitumor immune responses, thereby boosting the efficacy of current treatments, including those with and without ICB therapy. However, it is important to note that many of the treatments that have been investigated so far have been tested primarily in cell lines, where the concentrations used are often much higher than those suitable for clinical application. For example, statins are being evaluated at micromolar concentrations, whereas therapeutic doses of statins typically result in human serum concentrations ranging from 1 to 15 nM (115). Administration of such high doses of statins could result in undesirable side effects before the intended anti-tumor effects could be achieved. One possible solution to these challenges could be the development of advanced delivery systems, such as nanotechnology or liposomes, to more precisely target the delivery of cholesterol-lowering drugs. One possible solution to these challenges could be the development of advanced delivery systems, such as nanotechnology or liposomes, to more precisely target the delivery of cholesterol-lowering. There are currently some examples of this strategy (116, 117)

Another approach could be to explore possible synergies that amplify drug efficacy while maintaining optimal dosing. Another approach could be to explore possible synergies that amplify drug efficacy while maintaining optimal dosing. It would be valuable to investigate which cholesterol-lowering drugs synergize most effectively with radiotherapy or chemotherapy alone, or in combination with ICB therapies to increase immune antitumor activity, kill cancer cells, and improve patient survival. This includes determining optimal doses and treatment protocols, as well as elucidating the underlying biological mechanisms specific to each cancer type. Remarkably, current findings are limited to solid tumors, leaving a significant gap in the understanding of their effects in liquid cancers, which warrants further exploration.

Another important consideration is that different cell types vary in their ability to maintain cholesterol homeostasis. Immunosuppressive myeloid cells, such as monocytes, TAMs and neutrophils, as well as tumor cells, have significantly higher cholesterol levels and lipoprotein uptake rates than lymphocytes. Among lymphocytes, CD8^+^ T cells are particularly sensitive to cholesterol depletion, as low cholesterol levels hinder their proliferation and induce autophagy-mediated apoptosis. CD4^+^ T cells are less affected by cholesterol deficiency, demonstrating greater resistance to changes in cholesterol levels (118). With this in mind, research has shown that depletion of LXRβ in chimeric antigen receptor T cells (CAR-T) - thereby preventing cholesterol efflux - enhances their antitumor activity against solid tumors (118). Another potential therapeutic approach is the combination of CAR-T cell therapy with cholesterol-lowering drugs. Another potential therapeutic approach is the combination of CAR-T cell therapy with cholesterol-lowering drugs.

It is important to consider the possibility that cancer cells may develop resistance to cholesterol-lowering drugs. Several compensatory mechanisms can counteract these therapeutic interventions, allowing tumor cells to evade their effects. One of these mechanisms involves upregulation of SREBP2 or other pathways, such as PI3K/AKT and MYC, which enhance cholesterol biosynthesis. In addition, cancer cells may undergo metabolic reprogramming to adapt to cholesterol depletion. In this sense, alterations in sphingolipid metabolism may help cancer cells maintain lipid rafts and survival signaling, even in the absence of cholesterol (119). To counteract the inhibition of cholesterol synthesis, cancer cells may also increase LDL receptor (LDLR) expression to import more cholesterol from circulating lipoproteins. Another possible adaptation is to store esterified cholesterol in lipid droplets, which can be mobilized under cholesterol-limiting conditions. These and other alternatives must be thoroughly investigated and strategies developed to counteract them effectively.

Another interesting area of research that could be explored is the role of cholesterol in immune evasion during metastasis. This could occur through mechanisms such as altering the formation or function of immune synapses. Similarly, beyond the well-studied roles of oxysterols and cholesterol esters, other cholesterol-derived metabolites may also contribute significantly to tumor progression and immune evasion, representing an exciting avenue for future research.
